# Poor bladder compliance due to malacoplakia with xanthogranulomatous cystitis: A case report

**DOI:** 10.1097/MD.0000000000020852

**Published:** 2020-06-26

**Authors:** Ning Xiao, RongYu Tang, Bo Ge, HuaSheng Zhao, JianFeng Wang

**Affiliations:** a Department of Urology, The Second Affiliated Hospital of GuiLin Medical University, Guilin; b Continence Research Clinic, The Central Hosptial of Shaoyang, Shaoyang, China.

**Keywords:** malacoplakia, xanthogranulomas cystitis, lower urinary tract infection, bladder compliance, video urodynamic

## Abstract

**Rationale:**

Either malacoplakia or xanthogranulomatous cystitis (XC) is a rare chronic infection disease of urinary bladder, which often mimics bladder masses undifferentiated from malignance and results in severe lower urinary tract symptoms. The malacoplakia combined with XC is even rarer in the literature.

**Patient concerns:**

A 64-year-old female, who presented with nocturia, frequency of micturition, severe urgency with occasional urinary incontinence, and recurrent hematuria for >2 years, was diagnosed with azotemia and anemia. In addition, two 1.0 × 1.0 cm masses of bladder were detected by computer tomography.

**Diagnoses:**

Malacoplakia combined with xanthogranulomas cystitis was diagnosed histologically. Video urodynamic test showed poor bladder compliance (9 mL/comH_2_O), markedly decreased maximum bladder capacity (120 mL), and right vesicoureteral reflux at a low intravesical pressure level (25 cmH_2_O).

**Interventions:**

Transurethral resection of bladder masses was carried out after treatment of urinary infection by intravenous piperacillin-tazobactam. Oral Ciprofloxacin and Tolterodine were postoperatively used to prevent recurrent lower urinary tract infections and alleviate detrusor overactivity.

**Outcomes:**

The treatment did not alleviate azotemia, frequency, urgency with incontinence, and bilateral hydroureteronephrosis, but the patient refused to undergo bladder augmentation on account of her poor economic status.

**Lessons:**

Malacoplakia or/and xanthogranulomas cystitis may lead to poor bladder compliance and video urodynamic study should be considered in patients with refractory chronic lower urinary tract symptoms.

## 1 Introduction

Malacoplakia is a rare granulomatous disease that has been found to affect the genitourinary and gastrointestinal tracts, skin, lung, bone, and mesenteric lymph nodes.^[1]^ Furthermore, the urinary bladder is the most frequently involved organ.^[1]^ Grossly, malacoplakia presents as soft, yellow-brown plaques, and nodules, as well as bladder masses.^[2]^ Xanthogranulomatous cystitis (XC) also is a rare benign chronic inflammatory disease without defined etiology, and it is extremely difficult to clinically differentiate XC from bladder malignant tumor.^[3]^ There is only 1 case of a 9-year-old girl, who was diagnosed with spontaneous bladder perforation due to malacoplakia coexisting with XC, reported in English literature.^[4]^ So, we presented the second case of malacoplakia combined with XC leading to poor bladder compliance and refractory lower urinary tract symptoms (LUTS) in an old female patient.

## 2 Case report

**Figure 1:**
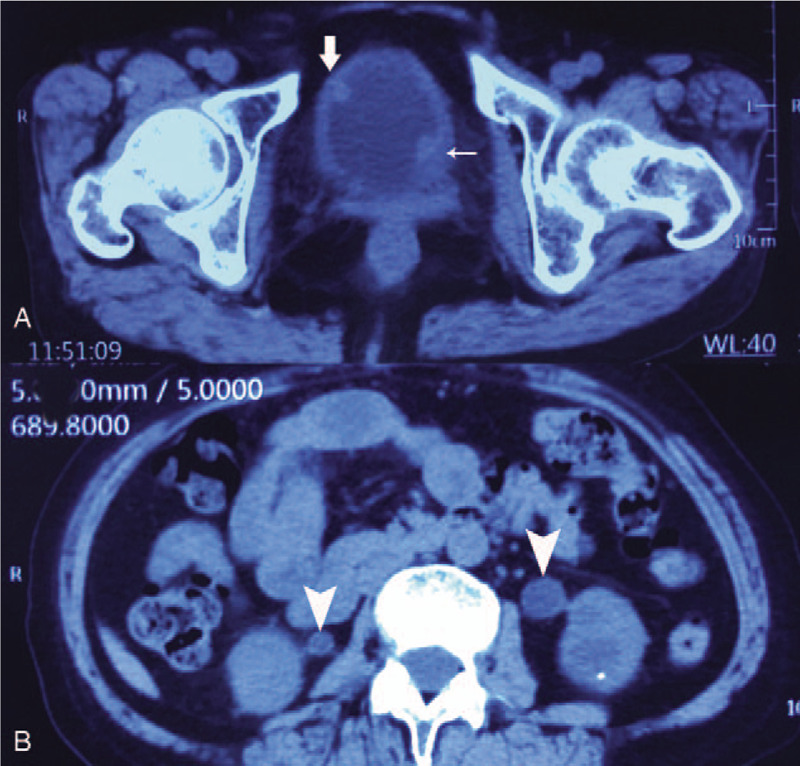
(A) Computer tomography (CT) demonstrated one 1.0 × 1.0 cm mass (thin white arrow) arising from left ureteral orifice, the other 1.0 × 1.0 cm mass (white thick arrow) at right wall of bladder, and extensive thickness of bladder wall. (B) Bilateral hydroureteronephrosis (white arrow head) was detected by CT.

**Figure 2:**
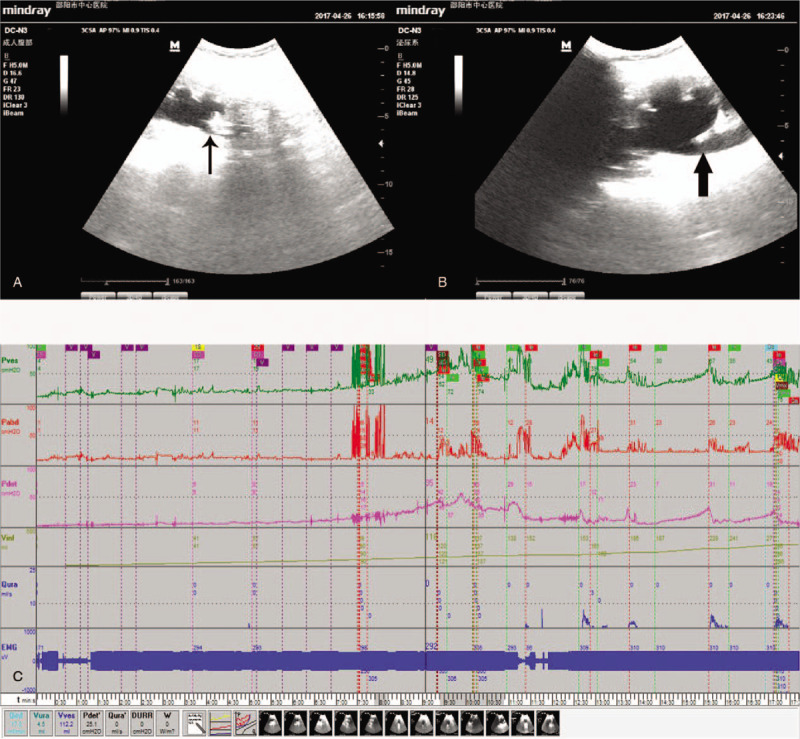
(A) Right hydroureteronephrosis (thin black arrow) was revealed at beginning of storage phase by sonography video urodynamic (VUD). (B) Aggravation of distention of right renal pelvis and ureter (thick black arrow), which indicated right vesicoureteral reflux (VUR), was detected at a low intravesical pressure (25 cmH_2_O). (C) The markedly decreased maximum bladder capacity (MBC) of about 120 mL (normal about 400 mL), at which the patient has a feeling of strong urgency desire to void, a poor bladder compliance of 9 mL/cmH_2_O (normal about 40 mL/comH_2_O), and detrusor overactivity was demonstrated by sonography VUD.

A 64-year-old woman presented with nocturia, frequency of micturition, severe urgency with occasional urinary incontinence, and recurrent hematuria for >2 years. Physical examination was unremarkable. She had undergone a bilateral percutaneous nephrolithotripsy for both renal calculi in April 2014, but denied any history of immunosuppressive diseases. Three weeks before transfer to our hospital, the patient underwent transurethral cystoscopy and biopsy, by which extensive mucosa hyperplasia and plaque necrotic lesions of bladder wall were found and XC was diagnosed pathologically. In our hospital, laboratory studies included normal hepatic and biochemical profiles, except for anemia (hemoglobin: 92 g/L; normal >120 g/L) and azotemia (creatinine: 208 μmol/L; normal <88 μmol/L) in the peripheral blood. Urine analysis showed plenty of red blood cells and white blood cells per high-power field. Urine cultures grew out *Klebsiella pneumonia*. Computer tomography (CT) demonstrated two 1.0 × 1.0 cm masses, of which one arising from left ureteral orifice and the other at right wall of bladder(Fig. 1A), irregularly extensive thickness of bladder wall (Fig. 1A), and bilateral hydroureteronephrosis (Fig. 1B). Sonography video urodynamic (VUD) test detected right vesicoureteral reflux (VUR) at a low intravesical pressure level (25 cmH_2_O) (Fig. 2A and B), the markedly decreased maximum bladder capacity (MBC) of about 120 mL (normal about 400 ml), at which the patient had a feeling of strong urgency desire to void, a poor bladder compliance of 9 mL/cmH_2_O (normal about 40 mL/cmH_2_O), and detrusor overactivity (DO) (Fig. 2C).

We informed the patient of treatments and risk, and obtained consent from patient with regard to publication of a case report in the future. Urinary infection was treated with intravenous piperacillin-tazobactam for 1 week and urine cultures were negative. Subsequently, transurethral resection of bladder masses was done cystoscopically. XC combined with malacoplakia, which is characterized by infiltration of histiocytes containing distinct basophilsic lysosomal inclusion bodies (Michaelis-Gutmann bodies), was ascertained by histological examination of resected specimen. The patient was discharged home and received oral Ciprofloxacin and tolterodine for 3 months to alleviate DO and prevent recurrent lower urinary tract infection (LUTI). After 3 months, the patients still complained of frequency and urgency with occasional incontinence, and azotemia was not improved obviously, but erythrocytes and leukocytes were not detected in urine examination. In follow-up, no significant improvement of bilateral hydroureteronephrosis was found by sonography. So, we suggested that the patient should undergo bladder augmentation due to poor bladder compliance and markedly decreased MBC, but she refused to our recommendation on account of her poor economic status.

## 3 Discussion

Both malacoplakia and xanthogranulomas are the chronic inflammatory diseases, associated with a spectrum of bacterial infections, that has been supposedly attributed to compromised immune system and can mimic a variety of infectious, inflammatory, and malignant disease.^[1,5]^ Malacoplakia most commonly affects urinary bladder and has a female preponderance. The pathognomonic Michaelis–Gutmann bodies have been reportedly deemed to be the result of chronic bacterial infection associated with impaired host defense and defective phagocytosis that leads to incomplete digestion of bacteria ingested by macrophages and subsequent mineralization and calcification of intracellular inclusions in chronically debilitated or immunosuppressed patients.^[6]^ Xathogranulomatomas is characterized microscopically by multinucleated giant cell, lipid-laden macrophages, and cholesterol crystals, but nothing about the disease has been clearly defined to date.^[7]^ Given the nonspecific characters of radiological and cystoscopic appearance in both conditions, the diagnosis should be made up histologically.

It is conceivable that nocturia, frequency, and severe urgency with occasional incontinence may be resulted from LUTI that derived from the nephrolithiasis diagnosed about 3 years ago. Poor bladder compliance and remarkably decreased MBC of the patient may arise from chronic bladder inflammation and subsequent detrusor fibrosis due to recurrent LUTI. A favorable outcome was reported by Kayigil et al^[8]^ after enterocystoplasty augmentation in female patients suffering from contracted bladder secondary to eosinophilic cystitis. Unlike the patient reported by Mukha et al^[2]^ who underwent a simple cystectomy with a short ileal conduit due to poor renal function and contracted bladder owing to malacoplakia, we suggested that the female patient should have undergone bladder augmentation on account of her preserved renal function (creatinine: 208 μmol/L) and maintenance of urinary continence. However, the recommendation of surgical procedure was refused by the patient due to her poor economic status. So, it should be kept in mind that early diagnosis and treatment of recurrent LUTI may prevent chronic fibrosis of detrusor that results in a poor bladder compliance and irretraceable LUTS.

Twenty-nine cases of XC have been reported in the literature since the first report by Wassiljew in 1932, whereas more than dozens cases of malacoplakia have usually been reported of associating with immunocompromised diseases or malignancy.^[6,9]^ The present case is the second report of patient suffering from malacoplakia combined with XC in the literature, but poor bladder compliance and impaired renal function of the present case were different from the first reported case of XC coexisting with malacoplakia leading to spontaneous perforation of bladder in a 9-year-old girl.^[4]^ Although the mechanism of that LUTI results in poor bladder compliance has not been definitely clarified,^[10]^ we inferred that chronic inflammation causes vascular changes, including endothelial hyperplasia, vascular occlusion, and perivascular fibrosis, which may lead to hypovascularity and hypoxia in bladder detrusor that contributed to collagen deposition and fibrosis resulting in decreased bladder compliance and capacity.

Malacoplakia or XC is diagnosed only by histological study and is commonly characterized by LUTS derived from chronic LUTI, which may lead to poor bladder compliance due to fibrosis of detrusor. Although rare incidence of malacoplakia coexists with XC, clinician should be aware that the disease may lead to irretraceable consequences and deterioration of patient's quality of life. Moreover, it is feasible that VUD should be carried out to evaluate the function of lower urinary tract for facilitation of accurate diagnosis and efficient treatment in patients suffering from refractory LUTS.

## Author contributions

**Conceptualization:** Ning Xiao, Bo Ge, HuaSheng Zhao, JianFeng Wang.

**Data curation:** Ning Xiao, RongYu Tang, Bo Ge.

**Formal analysis:** Ning Xiao.

**Funding acquisition:** Ning Xiao, JianFeng Wang.

**Investigation:** HuaSheng Zhao, JianFeng Wang.

**Writing – original draft:** Ning Xiao, RongYu Tang, Bo Ge.

**Writing – review & editing:** Ning Xiao.

## References

[1] Kogulan PK Smith M Seidman J Malakoplakia involving the abdominal wall, urinary bladder, vagina, and vulva: case report and discussion of malakoplakia-associated bacteria. Int J Gynecol Pathol 2001;20:403–6. 11603228 10.1097/00004347-200110000-00016

[2] Mukha RP Kumar S Ramani MK Isolated Malacoplakia of the bladder: a rare case report and review of literature. Int Urol Nephrol 2010;42:349–50. 19669585 10.1007/s11255-009-9624-z

[3] Wang Y Han XC Zheng LQ Xanthogranulomatous cystitis imitating bladder neoplasm: a case report and review of literature. Int J Clin Exp Pathol 2014;7:8255–8. 25550882 PMC4270612

[4] Sharma K Singh V Gupta S Xanthogranulomatous cystitis with malacoplakia, leading to spontaneous intraperitoneal perforation of the urinary bladder in a 9-year-old girl. BMJ Case Rep 2015;2015. 10.1136/bcr-2015-210786 PMC4550853 26272961

[5] Yu M Robinson K Siegel C Complicated genitourinary tract infections and mimics. Curr Probl Diagnost Radiol 2017;46:74–83. 10.1067/j.cpradiol.2016.02.004 26995297

[6] Lee SL Teo JK Lim SK Coexistence of malakoplakia and papillary urothelial carcinoma of the urinary bladder. Int J Surg Pathol 2015;23:575–8. 26194600 10.1177/1066896915595464

[7] Ong C Lee VK Mohamed Suphan N Xanthogranulomatous cystitis: a case report and clinicopathological review. Ann Acad Med 2013;42:301–3. 23842772

[8] Kayigil O Ozbagi T Cakar S Contracted bladder secondary to eosinophilic cystitis. Int Urol Nephrol 2001;33:341–2. 12092651 10.1023/a:1015201410322

[9] Hayashi N Wada T Kiyota H Xanthogranulomatous cystitis. Int J Urol 2003;10:498–500. 12941130 10.1046/j.1442-2042.2003.00669.x

[10] Yamada Y Minowada S Aruga T Contracted bladder developing after prostate brachytherapy. Int J Urol 2012;19:951–3. 22725658 10.1111/j.1442-2042.2012.03077.x

